# Self-Supervised Learning to Unveil Brain Dysfunctional Signatures in Brain Disorders: Methods and Applications

**DOI:** 10.34133/hds.0282

**Published:** 2025-08-05

**Authors:** Ying Li, Yanwu Yang, Yuchu Chen, Chenfei Ye, Ting Ma

**Affiliations:** ^1^ School of Electronic and Information Engineering, Harbin Institute of Technology (Shenzhen), Shenzhen, China.; ^2^ Department of Psychiatry and Psychotherapy, Tübingen Center for Mental Health, Tübingen, Germany.; ^3^ School of Biomedical Engineering, Harbin Institute of Technology (Shenzhen), Shenzhen, China.; ^4^ International Research Institute for Artificial Intelligence, Harbin Institute of Technology (Shenzhen), Shenzhen, China.; ^5^ Peng Cheng Laboratory, Shenzhen, China.

## Abstract

**Importance:** Precisely decoding brain dysfunction from high-dimensional functional recordings is crucial for advancing our understanding of brain dysfunction in brain disorders. Self-supervised learning (SSL) models offer a transformative approach for mapping dependencies in functional neuroimaging data. Leveraging the intrinsic organization of brain signals for comprehensive feature extraction, these models enable the analysis of critical neurofunctional features within a clinically relevant framework, overcoming challenges related to data heterogeneity and the scarcity of labeled data. **Highlight:** This paper provides a comprehensive overview of SSL techniques applied to functional neuroimaging data, such as functional magnetic resonance imaging and electroencephalography, with a specific focus on their applications in various neuropsychiatric disorders. We discuss 3 main categories of SSL methods: contrastive learning, generative learning, and generative-contrastive methods, outlining their basic principles and representative methods. Critically, we highlight the potential of SSL in addressing data scarcity, multimodal integration, and dynamic network modeling for disease detection and prediction. We showcase successful applications of these techniques in understanding and classifying conditions such as Alzheimer’s disease, Parkinson’s disease, and epilepsy, demonstrating their potential in downstream neuropsychological applications. **Conclusion:** SSL models provide a scalable and effective methodology for individual detection and prediction in brain disorders. Despite current limitations in interpretability and data heterogeneity, the potential of SSL for future clinical applications, particularly in the areas of transdiagnostic psychosis subtyping and decoding task-based brain functional recordings, is substantial.

## Introduction

Elucidating the spatiotemporal dynamics of brain functional reorganization in network neuroscience has advanced translational medical applications in brain disorders. This includes uncovering pathophysiological processes, enabling early identification, and facilitating therapeutic interventions for these conditions. Nevertheless, precisely decoding brain dysfunction from individual high-dimensional functional recordings, particularly those obtained through functional magnetic resonance imaging (fMRI) and electroencephalography (EEG), still presents a considerable challenge. This is due to the inherent complexity of spatiotemporal functional activities within the brain’s multi-scale network architecture. Despite recent technical advancements in applying deep learning to decode brain dysfunction in brain disorders, the generalizability of these models is often limited. This limitation mainly stems from their reliance on supervised learning paradigms, which require sample-level annotations that assign specific brain disorders or behavioral phenotypes to individual data points [1]. Data heterogeneity, labeling discrepancies, and brain disorder diversity across imaging sites hinder the development of accurate, context-wide neuroimage decoding models [2,3].

Self-supervised learning (SSL) has emerged as a promising tool for functional neuroimaging decoding due to its ability to leverage vast amounts of unlabeled data [4]. Annotating brain data is often time-consuming, costly, and further complicated by the inherent temporal variability of neurological conditions. In contrast to conventional supervised learning approaches that are heavily dependent on large annotated datasets, SSL employs pretext tasks like contrastive learning and generative reconstruction to learn directly from unlabeled data. This is particularly important in neuroscience, where the scarcity of labeled data presents a marked challenge [5]. By extracting intrinsic characteristics from high-dimensional neural signals, the SSL model captures the spatiotemporal patterns of brain phenotypes, allowing transferability to external datasets and new tasks. This attribute positions SSL as a transformative paradigm within the realm of functional neuroimaging research, offering a more scalable and effective tool to support clinical applications. Recent developments in SSL models, trained on diverse neuroimage datasets [5–8], encompassing generative learning, contrastive learning, and generative-contrastive frameworks [9], have shown remarkable potential in downstream neuropsychological applications, including disease detection and outcome prediction [10–13]. While some literature reviews have discussed the technical merits and challenges of SSL [9,14,15], a thorough overview of SSL-based medical applications in neuropsychological diseases remains to be established.

This paper provides a thorough examination of the application of SSL techniques for decoding brain dysfunction in the context of brain disorders. It begins with an overview of the foundational SSL methodologies and their significance in brain network computation. Key medical applications of SSL in addressing challenges such as data scarcity, multimodal integration, and dynamic network modeling in brain disorders are then discussed. The paper concludes by highlighting the current limitations in SSL adoption for brain network analysis and proposing potential directions for future research to overcome these challenges.

## SSL Models

**Fig. 1.**
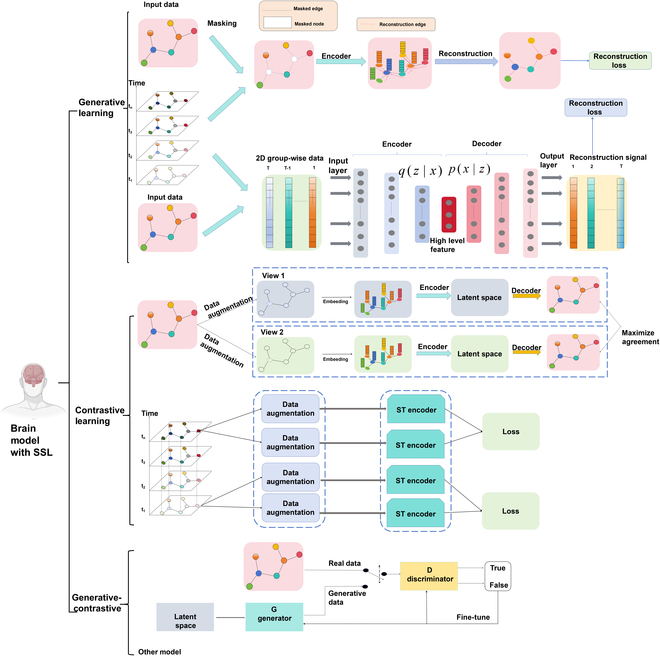
Overview of the typical SSL pipeline for neuroimaging data analysis. The top represents the brain network pipeline, where raw neurological data are systematically processed to extract meaningful representations. The bottom highlights the core self-supervised model, comprising an encoder–decoder architecture. These refined representations are then utilized for downstream tasks, such as disease categorization, detection, and prediction. The model’s bidirectional learning flow ensures robustness and adaptability across diverse neuroimaging datasets.

**Fig. 2.**
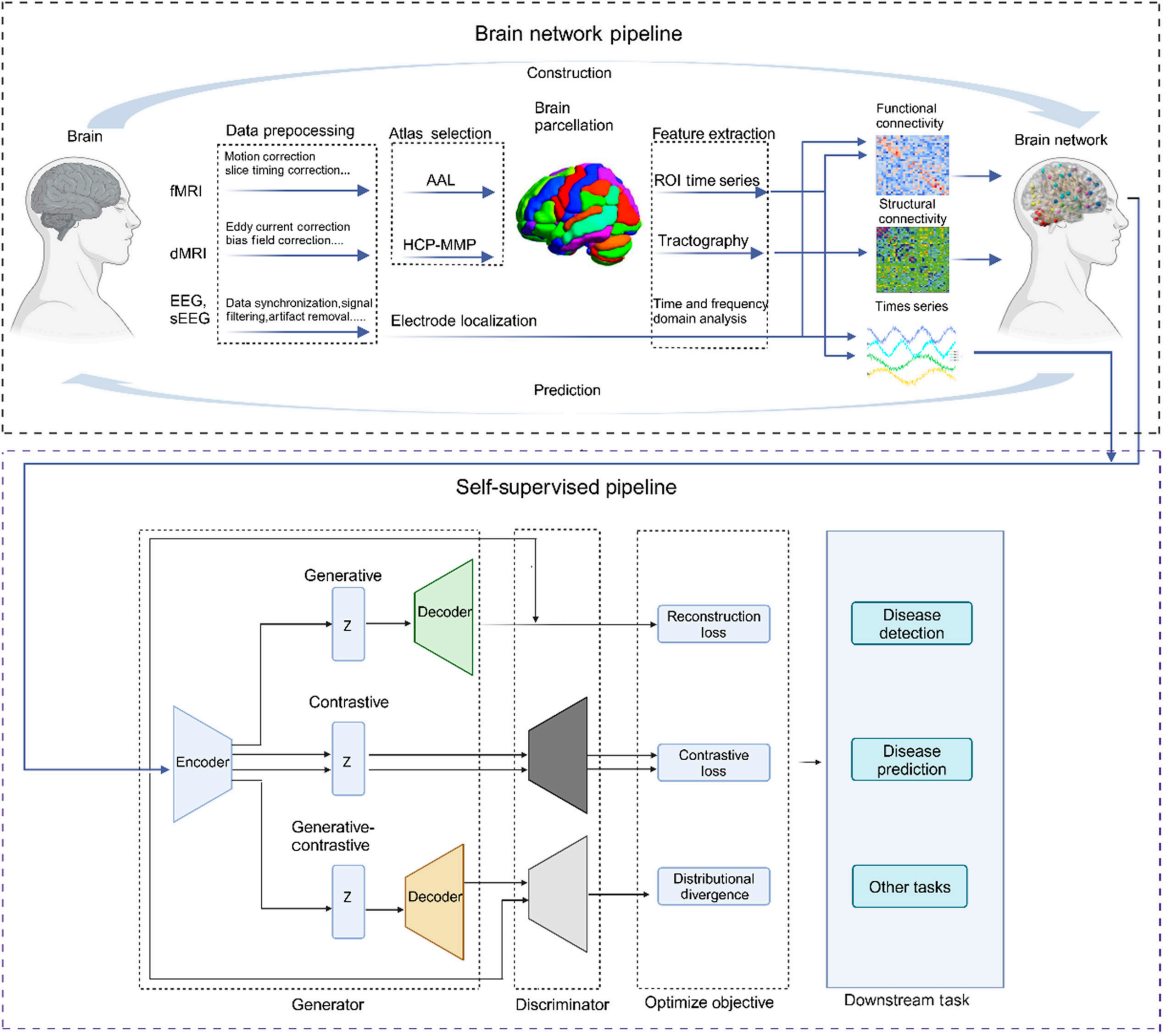
The primary learning strategies within SSL models in neuroimage-based medical applications. In contrastive learning, the graph-based approach generates augmented views of brain graphs to maximize view similarity through encoders and decoders, while the spatiotemporal-based approach focuses on leveraging temporal neural signals for similar contrastive objectives. Generative learning includes a mask-based method, which reconstructs randomly masked brain regions to minimize reconstruction loss, and a VAE-based method, where neural imaging data are encoded and reconstructed to learn global patterns. Last, generative-contrastive learning combines generative modeling, such as GANs, with contrastive learning to capture intrinsic brain representations.

Brain disease detection based on neuroimaging has been a challenge, attributed to the intricate reorganization of the brain network and the diverse neuropathological manifestations [16]. By processing high-dimensional functional neuroimaging data, SSL models hold great promise for learning representations of complex neural activity, thereby facilitating downstream tasks. A general pipeline of SSL in neuropathological applications is illustrated in Fig. 1. SSL models have recently demonstrated significant potential in the field of neuroimaging analysis. The core idea of SSL is to leverage vast amounts of unlabeled data for pretraining, learning general data representations to overcome the challenge of data annotation scarcity. The pretext task is a crucial component within SSL models. The pretext task, also known as an auxiliary task or pretraining task, is a core component of SSL. It is designed to create an artificial, easily solvable supervised learning task that uses unlabeled data to train a model, enabling it to learn general representations relevant to the data’s intrinsic characteristics. This learned representation then provides a foundation for downstream real-world tasks. In the field of neuroimaging, data annotation is costly, while unlabeled data are abundant. The introduction of pretext tasks allows us to fully utilize this unlabeled neuroimaging data to pretrain models and learn general representations of brain networks or brain activity patterns. These representations can be effectively transferred to various downstream tasks, such as brain disease classification and disease severity regression, improving model performance on these tasks and enhancing model generalizability across different datasets. In the field of neuroimaging, the design of pretext tasks also needs to fully consider the characteristics of neuroimaging data, such as high dimensionality, spatiotemporal dependency, and graph structure. In the following sections, we will introduce several typical SSL techniques, including contrastive learning, generative learning, and generative-contrastive learning, as shown in Fig. 2.

### Contrastive learning

Contrastive learning aims to enhance the similarity of positive pairs of samples while reducing the similarity of negative pairs [17]. By embedding high-dimensional data into latent spaces and aggregating similarities and alienating dissimilarities, contrastive learning models can effectively capture complex brain activity patterns through latent spatial representations, rendering them suitable for brain disorder detection. In the context of neuroimaging data, one can define positive pairs as patient data from the same brain region or contiguous time domains, and negative pairs as data from different brain regions or noncontiguous time domains [18]. Currently, contrastive learning has been widely used in processing human brain signals, including EEG and fMRI scans [19–21].

#### Graph-based contrastive learning

Brain network data can be intuitively represented as graphs, where nodes correspond to brain regions or electrode positions, and edges capture functional or structural connectivity [22]. This graph-based representation allows for the modeling of complex brain networks and facilitates the application of advanced methods such as graph-based contrastive learning (GBCL) [23], which has shown promise in uncovering meaningful representations from brain data. Graph-based neural networks (GNNs), as a powerful deep learning framework for processing graph-structured data, have demonstrated remarkable capabilities in modeling non-Euclidean data structures such as graphs. However, the inherent challenges of data labeling in these spaces necessitate the development of innovative solutions. Recent advancements in GBCL combine the representational power of GNNs with the efficiency of SSL to extract meaningful features from brain connectivity maps [24]. Within the framework of GNNs, various advanced models have been designed to tackle specific challenges in brain network analysis, including effective feature representation, noise reduction, and dynamic brain state modeling. While graph convolutional networks (GCNs) are primarily used for supervised learning tasks, their core ability to capture local and global features in graphs has inspired extensions into self-supervised frameworks, such as contrastive learning or graph autoencoders, which are better suited for tasks involving large-scale unlabeled brain data. For example, the contrastive FC graph learning (CGL) framework [25] enhances connectivity pattern representations using spectral convolution to model complex interactions across brain regions. In CGL, the pretext task involves contrastive learning on functional connectivity (FC) graphs, where the model defines positive pairs as FC graphs derived from nonoverlapping region of interest (ROI) time series of the same subject and negative pairs as FC graphs from different subjects. This task encourages the model to learn representations that capture subject-specific connectivity patterns by maximizing the similarity between positive pairs and minimizing it between negative pairs, using a contrastive loss function optimized over spectral graph convolutions. Additionally, a dynamic population map is introduced, adapting network characteristics over time based on patient similarity, thereby improving the clustering of similar cases. GATE (graph CCA for temporal SSL) [26] reinterprets contrastive learning for fMRI analysis by leveraging canonical correlation analysis (CCA) as a novel alignment mechanism. Through dynamic FC-based augmentations—step window augmentation (S-A) and multi-scale window augmentation (M-A)—GATE generates diverse yet correlated views of blood oxygen level-dependent (BOLD) signals, serving as positive pairs in the contrastive framework. The pretext task in GATE involves generating these augmented views of BOLD signals as positive pairs, contrasting them against views from different subjects or uncorrelated segments (negative pairs). The CCA-based loss maximizes the similarity between these embeddings while regularizing feature decorrelation to prevent collapse. Similarly, CMV-CGCN [27] integrates FC and higher-order functional connectivity (HOFC) features within a contrastive multi-view learning framework. By defining positive pairs (FC and HOFC of the same subject) and negative pairs (FC or HOFC from different subjects), the model employs a contrastive loss to maximize similarity between positive pairs while minimizing similarity between negative pairs. In addition to incorporating graph convolutional neural networks, MeTSK [28] employs a novel meta-learning strategy that integrates SSL with a contrastive learning framework on graphs, facilitating effective knowledge transfer across domains. In the source domain, a graph contrastive loss is used to align embeddings from different temporal views of the same subject (positive pairs) while distinguishing them from embeddings of different subjects (negative pairs). Concurrently, the target-specific task is optimized using a bilevel meta-learning framework, where the inner loop adapts to the target task, while the outer loop updates transferable graph representations. The fusion of advanced feature extraction and contrastive learning techniques enhances the flexibility and general applicability of models in brain network analysis, significantly benefiting neuroimaging data processing. For instance, to address the issue of noise, diffusion learning has been incorporated in some models. DGCL [29] introduces the brain region-aware module to dynamically adjust population maps through diffusion processes, reducing noise and eliminating disease-related connections to enhance brain network construction. Similarly, BrainNet [30] incorporates hierarchical graph diffusion learning to simulate the spread of epileptic waves from stereoelectroencephalography (SEEG) data. Through the use of bidirectional contrastive predictive coding (BCPC), it aligns temporal embeddings by contrasting related and unrelated segments, ensuring that the learned representations capture essential dynamics. In BrainNet, the pretext task involves contrasting related temporal segments (positive pairs) against unrelated segments (negative pairs) within SEEG data, enabling the model to learn representations that reflect the temporal dynamics of epileptic wave propagation, which are critical for understanding and predicting seizure patterns.

In summary, graph-based SSL leverages the structural characteristics of graph data to efficiently extract both local and global features from brain networks without requiring labeled data, providing robust support for complex brain network representation. The incorporation of graph-specific properties enhances model adaptability and robustness, ensuring consistency in feature representation by reducing noise and eliminating irrelevant connections. Techniques such as contrastive learning optimize the representation of nodes and edges, resulting in improved accuracy and generalization in detection and prediction tasks. These advantages establish graph-based SSL as a critical approach for analyzing graph-structured data in neuropsychological disease research.

#### Spatiotemporal-based contrastive learning

Effectively capturing spatial and temporal dependencies is crucial for the integration of multichannel neurophysiological time-series data regarding complex functional activity of the brain [31,32]. A dual-perspective approach that integrates spatial and temporal patterns allows the SSL model to identify dynamic changes in brain networks, thereby enhancing its discriminative or predictive capacity for brain diseases [33,34]. While traditional methods in neurosignal analysis often rely on static representations or simplistic aggregation of temporal features, they fail to capture the intricate spatiotemporal dynamics and evolving patterns within brain networks. Advanced models [35–37] have shifted toward leveraging time-series data, enabling the analysis of dynamic connectivity changes and long-range dependencies. To further enhance this capability, spatiotemporal-based contrastive learning frameworks have emerged, offering a more robust approach to disentangle temporal variations and spatial relationships, thereby addressing the constraints imposed by static and linear methods. For example, spatiotemporal hierarchical enhancement-based contrastive learning (ST-HACL) [38] constitutes an advanced framework designed to improve spatiotemporal neurosignal analysis, combining contrastive learning principles with a GNN architecture. The InfoNCE objective function serves as the foundation, enabling SSL through the optimization of feature representations based on contrastive samples. The model distinguishes positive and negative sample pairs, which are critical to enhancing its ability to capture dynamic temporal relationships. Positive samples are created through augmentations of the same brain network, such as temporal window cropping or signal compression. In contrast, negative samples originate from augmented brain networks of different subjects. Hierarchical augmentation strategies tailored to brain network construction ensure the generation of high-quality contrastive samples, which strengthens the model’s ability to learn intricate spatiotemporal dependencies within dynamic brain signals. By introducing these complex pretext tasks, ST-HACL models achieve superior performance in learning long-range temporal dependencies and spatial interactions across different brain regions. Traditional approaches, including recurrent neural networks (RNNs) and spatiotemporal deep infomax (ST-DIM) [39], provide foundational insights that ST-HACL extends to achieve superior robustness and accuracy in predicting neurological disease progression. However, the pretext tasks in ST-HACL, through their ability to generate augmented views of the brain network and encourage the model to learn temporal and spatial relationships, elevate the model’s performance significantly beyond what is achievable by traditional methods.

### Generative learning

Generative learning addresses the challenge of annotation scarcity in neurophysiological signal decoding by synthesizing features that closely resemble those found in real data, through the process of learning the underlying distribution of these samples [40]. In this study, we mainly focus on 2 primary generative learning models applied in neuroimaging data, specifically variational autoencoders (VAEs) and mask-based learning (MAE). VAEs excel in latent representation and facilitate tasks like anomaly detection in brain imaging through efficient reconstructions [41]. In contrast, MAE complements generative goals by reconstructing missing or corrupted data, ensuring data integrity [42]. These approaches are particularly valuable in neuroimaging, where labeled data are often limited, and generative models can mitigate this by producing synthetic samples that enhance downstream tasks such as disease classification. However, their reliance on reconstruction objectives may prioritize low-level details over high-level features critical for classification, a limitation noted in generative SSL [9].

#### VAE-based generative learning

By leveraging probabilistic modeling of brain network structures, VAEs are capable of extracting disentangled latent representations of neural information. For instance, the deep causality variational autoencoder (CVAE) [43] model extends the traditional VAE framework by introducing a causal layer, enabling the direct inference of causal relationships between brain regions from time-series fMRI data. This approach bypasses the restrictive constraints of conventional methods, such as acyclic graph structures, while simultaneously reconstructing brain network structures and capturing spatiotemporal dynamics. Similarly, the deep variational autoencoder (DVAE) [44] model employs variational inference to uncover underlying structures within high-dimensional MRI data, generating interpretable representations of brain activity and enables the extraction of generalized features, enhancing the robustness and generalizability of brain network analysis. DynaMorph [45] advances the analysis of dynamic brain morphology through a vector quantization variational autoencoder (VQ-VAE) framework. By integrating temporal regularization, DynaMorph ensures smooth transitions across various brain states, facilitating the examination of brain dynamics in diverse contexts. This simultaneous learning of latent characterizations enables the model to delineate and analyze complex spatiotemporal changes in cellular brain networks, promoting a deeper understanding of time-evolving brain structures. Beyond standalone VAE-based frameworks, hemispherically separated cross-connected group aggregate learning (HCAL) [46] combines the strengths of VAEs and generative adversarial networks (GANs) in a VAE-GAN hybrid architecture. HCAL, tailored for neurodegenerative disease analysis, synthesizes diverse and realistic structural connectivity matrices by emphasizing both intra- and interhemispheric connections. Its hemispherical dissociation generator adeptly captures local and global topological features, and a connection-aware discriminator stabilizes adversarial training, thereby enriching the structural connectivity data essential for brain network analysis. In summary, VAE-based models have demonstrated exceptional efficacy in brain network analysis, particularly in capturing complex spatiotemporal dynamics within brain regions. Through innovative frameworks and hybrid architectures, these models provide robust tools for addressing challenges such as data scarcity, overfitting, and the inherent variability of brain networks. Their strength lies in modeling complete data distributions, making them ideal for tasks requiring reconstruction, such as anomaly detection in brain imaging. However, for disease classification tasks like identifying neurodegenerative patterns, their focus on reconstruction may capture extraneous details irrelevant to discriminative features, potentially reducing performance compared to contrastive methods. Additionally, their computational complexity can hinder scalability when applied to large-scale neuroimaging datasets.

#### Mask-based generative learning

Mask-based models tackle the challenges posed by the complexity of brain connectivity and noise uncertainty in traditional brain network analysis, which often result in incomplete representations. By strategically masking regions or connections during training, these models are better equipped to learn deeper and more meaningful representations of brain networks. For instance, the BrainMAE [47] model utilizes a mask autoencoding framework specifically designed for fMRI data, where the key innovation lies in the region-aware graph attention mechanism, which focuses on the interactions between brain regions while reconstructing masked areas, and enhances the model’s ability to process noisy fMRI data, leading to the extraction of robust and interpretable brain signal representations. Similarly, EAG-RS [48] employs a random seed-based network masking approach, masking ROI to force the model to learn nonlinear relationships between functional connections. By prioritizing higher-order functional connections and incorporating hierarchical correlation propagation (LRP), this method ensures that the reconstructed connections are indispensable for accurately recovering the masked functional connections. Overall, the fusion of masking strategies and advanced graph-based techniques underscores the importance of mask-based models as a robust tool for tackling challenges in brain network analysis. Models excel in denoising and enhancing data integrity, which is crucial for handling noisy neuroimaging data and can support disease classification by reconstructing disease-relevant connectivity patterns, such as those altered in disorders like epilepsy. Nevertheless, their effectiveness heavily depends on the masking strategy; poorly designed masks may fail to emphasize critical features, leading to suboptimal representations for downstream tasks. Moreover, unlike contrastive methods that directly optimize class separation, mask-based approaches may struggle to prioritize discriminative features, potentially limiting their utility in fine-grained classification tasks.

### Generative-contrastive learning

Generative-contrastive SSL leverages the advantages of generative models and contrastive learning for robust representation learning. It typically includes 2 principal components: a generator and a discriminator. The generator produces synthetic data that approximate the true data distribution, while the discriminator learns to distinguish between real and generated samples. Here, we will introduce advantages of some generative-contrastive SSL models applied in neuroimaging data.

#### GAN-based generative-contrastive learning

Among generative-contrastive methods, GAN stands out as the most notable model [49]. By generating and contrasting synthetic brain networks or functional activities, this architecture tackles key challenges in brain network analysis, including the scarcity of large labeled datasets and the variability inherent in brain connectivity. This approach can particularly optimize the use of small-sample datasets, bolstering model robustness and generalizability. Furthermore, GANs support tasks such as cross-modal synthesis and data completion, which can expedite training processes and enhance the accuracy of predictions in neurological disease diagnosis. For instance, in the context of disease classification, such as distinguishing Alzheimer’s disease (AD) from healthy controls, GANs can generate synthetic brain connectivity patterns to augment limited labeled data, thereby improving the model’s ability to identify subtle disease-specific features that might otherwise be obscured by data scarcity [9]. However, their effectiveness depends on the stability of training, as GANs are prone to collapse without careful tuning. For instance, graph-based conditional generative adversarial networks (GC-GANs) [50] adopt a conditional GAN framework that incorporates additional information, such as node or graph labels, to guide the generation process, which ensures that the synthetic graph structures remain consistent with their real counterparts. Furthermore, a class-aware discriminator enhances the diversity and quality of generated outputs, mitigating the issue of data scarcity while preserving the global and local topological characteristics of brain networks. In GraphGAN++ [51], the graph generator employs Wasserstein generative adversarial networks (WGANs) [52] to stabilize the training process and prevent mode collapse. Additionally, a 3-stage learning framework, combined with a topological loss function, reduces uncorrelated multi-graph clustering and noise-related issues in brain network generation. By integrating GCNs with α-GAN, the α-GCNGAN [53] framework uses graph variational autoencoders (GVAEs) [54] to model the intrinsic structure of brain networks. The encoding discriminator further ensures that the posterior distribution of the latent space aligns with the prior, achieving more accurate graph generation. This precision in modeling brain network topology can be particularly beneficial for disease classification tasks, where capturing fine-grained connectivity differences—such as those between healthy and diseased states—is critical. Yet, the computational complexity and potential for overfitting to generated samples may limit its scalability across diverse neuroimaging datasets [9].

#### Other generative-contrastive learning

BrainMass [55], a novel generative-contrastive learning model, captures individual brain activity patterns across over 30 datasets, demonstrating strong generalizability in identifying various brain disorders. It integrates the masked region module (MRM) and latent representation alignment (LRA) module to enable simultaneous generative-contrastive learning. The MRM in BrainMass randomly masks brain regions and uses the remaining features to reconstruct the masked areas, thereby strengthening inter-regional connections and maintaining local network properties. Meanwhile, the LRA module regularizes augmented brain networks from the same subjects, ensuring similarity in latent embeddings despite pseudo-FC enhancements. This dual-module design balances generative learning through feature reconstruction with contrastive alignment of network representations, facilitating the accurate extraction of disease-specific biomarkers. Empirical evidence indicates that the dual-module architecture excels in terms of generalizability and adaptability, in the context of disease discrimination across various conditions. Compared to purely contrastive methods like SimCLR [17], BrainMass’s generative component enhances its ability to model complex brain activity patterns, making it particularly suitable for diseases with heterogeneous manifestations, such as schizophrenia or epilepsy. However, its reliance on reconstructing masked regions may introduce biases if the masking strategy does not adequately reflect disease-relevant variability, a limitation noted in generative methods by Liu et al. [9]

Contrasting BrainMass’s focus on single-modal masking and attention, SSL models encompassing decomposed-VAE module specialize in multimodal fusion and decomposition, enhancing the understanding of brain network’s structural-functional interplay. For instance, a novel SSL model, named brain structure-function fusion-representation learning (BSFL), is introduced to efficiently derive integrated representations from structural and functional MRI data for the detection of mild cognitive impairment (MCI). Specifically, the generative module, implemented through variational graph autoencoders, decomposes the feature space of multimodal MRI data into shared and modality-specific representations. These representations are reconstructed to retain unimodal information, while the fusion of decomposed features generates unified brain networks, thereby ensuring structural-functional complementarity. Besides, the contrastive learning module utilizes a uniform-unique contrastive loss function to maximize the separation between unique representations within each modality while minimizing the distance between shared representations across modalities. This synergistic design enhances the accuracy of feature decomposition and effectively captures complementary, disease-specific connectivity patterns, leading to a significant improvement in the prediction of brain abnormalities associated with MCI.

## Medical Applications of SSL

SSL models demonstrate a remarkable advantage by harnessing extensive unlabeled datasets to learn the intrinsic network reorganization signatures associated with brain disorders, thus paving the way for innovative tools in disease screening, diagnostic detection, and prognostic forecasting. To comprehensively review the applications of SSL in brain functional impairments of brain disorders, we conducted a systematic literature search and screening. The databases searched include PubMed, Web of Science, and Scopus, covering the period from 2019 to 2024. The search keywords used were self-supervised learning, contrastive learning, generative learning, brain, fMRI, EEG, and brain disorders. The review was limited to literature in the English language. The titles and abstracts of all retrieved articles were examined. For those works that appeared to be potentially relevant, the full texts were accessed and retrieved. Duplicate publications were excluded. Following a rigorous literature screening spanning the past 5 years, a selection of 26 related literatures has been identified and listed in Tables 1 to 3. We will summarize these medical applications as below.

**Table 1.** Literatures of brain activity decoding using generative learning in brain disorders

Index	References	SSL method	Data type	Data modality	Dataset	Sample size	Downstream tasks	Performance metrics	Model architecture
1	Alfakih et al. [43]	Deep CVAE (deep causal VAEr)	BOLD	fMRI	ADNI, ABIDE	HC: 784 ASD: 287 MCI: 673	Disease classification	ADNI dataset: accuracy: 0.756 ABIDE dataset: accuracy: 0.714	It comprises an encoder, a decoder. Both the encoder and decoder consist of 3 hidden layers, each containing 100 ReLU units. This model builds upon the concept of the linear structural equation model (SEM) and relaxes the acyclic constraint often found in other causal inference methods.
2	Qiang et al. [44]	DVAE (deep VAE)	FC	fMRI	ADHD-200	HC: 266 ADHD: 281	Disease classification	ADHD-200 dataset: accuracy: 0.725	The main architecture is a DVAE with an encoder consisting of 3 convolutional layers and a decoder with 3 deconvolutional layers.
3	Wang et al. [92]	ACVAE (adversarial conditional VAE)	BOLD	fMRI	OASIS-3	HC: 1,476 AD: 21	Disease classification	OASIS-3 dataset: AUC: 0.912 ADNI dataset: AUC: 0.901	The main architecture is CVAE with an encoder and a decoder, both utilizing fully connected layers. The model also incorporates an adversarial learning component.
4	Choi et al. [93]	ST-JEMA (spatiotemporal joint embedding MAE )	BOLD	fMRI	HCP, ABCD	HC: 372 ADHD: 512	Age regression; gender classification	ABIDE dataset: accuracy: 0.718 PPMI dataset: accuracy: 0.803	It consists of an online encoder and a target encoder. Both encoders, based on GNNs and a temporal encoder, process dynamic functional connectivity matrices. The online encoder takes a masked input sequence, and its output is fed into a predictor network to predict the representation of the unmasked parts processed by the target encoder.
5	Yang et al. [47]	BrainMAE (brain MAE)	BOLD	fMRI	HCP, ABCD	HC: 897 Aging dataset participants: 725	Gender classification; age and behavior prediction	ABIDE dataset: accuracy: 0.746 HCP dataset for age regression: MAE: 2.7 HCP dataset for gender classification: accuracy: 0.941	BrainMAE is a transformer-based masked autoencoder. Its encoder uses a region-aware graph attention mechanism to process masked fMRI time series, and the decoder reconstructs the original signals.
6	Choi et al. [94]	ST-MAE (spatiotemporal-MAEr)	FC	fMRI	UKB, ABIDE, ADHD200, ABCD, HCP	HC: 40,913 ASD: 884 ADHD: 669	Disease classification	ABIDE dataset: accuracy: 0.734 HCP dataset for age regression: MAE: 2.88 HCP dataset for gender classification: accuracy: 0.932	ST-MAE is a spatiotemporal masked autoencoder. Its encoder consists of a GCN for spatial features and a Transformer for temporal features. The decoder is an MLP that reconstructs masked functional connectivity matrices.
7	Jung et al. [48]	EAG-RS (explainability-guided ROI selection)	FC	fMRI	ABIDE	HC: 462 ASD: 418	Disease classification	ABIDE dataset: accuracy: 0.93	For inter-regional relation learning, EAG-RS uses a multi-layer perceptron (MLP). One MLP is trained for each ROI to predict its masked connections based on the rest of the brain network.
8	Wang et al. [63]	GCN_SSL (GCN with self-supervised learning)	FC	fMRI	ADNI, ABIDE	HC: 174 ASD: 79 MCI: 68	Disease classification	ADNI dataset: accuracy: 0.808 ABIDE dataset: accuracy: 0.745	The architecture is a 2-layer GCN trained with self-supervised contrastive learning. It learns by ensuring similar embeddings for different corrupted views of the same brain network, and the resulting embeddings are used for disease classification
9	Wen et al. [72]	BrainGSL (graph self-supervised learning)	FC	fMRI	ABIDE, NMU	HC: 714 ASD: 403 MDD: 151 BD: 126	Disease classification	ABIDE dataset: accuracy: 0.713	The main network architecture consists of a local topological-aware encoder based on a GCN and a node-edge bi-decoder. The GCN encoder takes a partially masked brain network as input and learns node embeddings. The decoder then uses these embeddings to reconstruct the masked edges of the original brain network. The framework also includes a signal representation learning module (using LSTM for BOLD signals) and a classifier.

**Table 2.** Literatures of brain activity decoding using contrastive learning in brain disorders

Index	References	SSL method	Data type	Data Modality	Dataset	Sample size	Downstream tasks	Performance metrics	Model architecture
10	Cui et al. [28]	MeTSK (meta transfer with self-supervised knowledge)	FC	fMRI	HCP, ADHD-200	HC: 143 ADHD: 102	Disease classification	ADHD dataset: accuracy: 0.721	The main network architecture is a 2-layer GCN. It takes a brain network as input and outputs a graph-level embedding.
11	Chen et al. [30]	BrainNet (BCPC + graph diffusion learning)	SEEG time series, FC	SEEG	SEEG dataset	Epileptic patients: 10	Disease classification	SEEG dataset: AUC: 0.922	BrainNet uses GCNs to learn from brain connectivity graphs that are constructed using a hierarchical graph diffusion learning process on SEEG data.
12	Zong et al. [29]	DGCL (diffusion-based graph contrastive learning)	SC	dMRI	ADNI, ABIDE	HC: 87 EMCI: 135 LMCI: 63 AD: 64	Disease classification	ABIDE dataset: F1 score: 0.714	DGCL’s main architecture is a 2-layer GAT that learns representations from constructed brain networks.
13	Shi et al. [95]	HebrainGNN (heterogeneous brain network GNN)	Heterogeneous graph representations	fMRI, MRI	ADNI, private cohort	HC: 250 MCI: 85 EMCI: 84 LMCI: 35 AD: 58	Disease classification	OH dataset: accuracy: 0.738 ADNI dataset: accuracy: 0.773	HebrainGNN’s main architecture is a 2-layer heterogeneous GNN designed to process brain networks with different types of nodes (hemispheres) and edges (intra-/interhemispheric).
14	Meng et al. [96]	CvFormer (cross-view transformer with contrastive learning)	FC	fMRI	ADNI, ABIDE	HC: 1,112 AD: 34 EMCI: 54 LMCI: 38	Disease classification	ADNI dataset: accuracy: 0.891 ABIDE dataset: accuracy: 0.959	CvFormer uses a dual-branch architecture with Transformer encoders to process ROI time series and functional connectivity matrices separately, and a cross-view module to integrate information between the 2 branches.
15	Cai et al. [64]	MBrain (multi-channel contrastive predictive coding)	SEEG time series, FC	SEEG, EEG	In-house dataset	Annotated seizures: 3,050 Seizure types: 8	Disease classification	SEEG dataset (subject dependent experiment): F1 score: 0.465 EEG dataset (domain generalization experiment): AUC: 0.779	MBrain is an MAE where both the encoder and decoder are based on the Transformer architecture.
16	Zhu et al. [27]	CMV-CGCN (contrastive multi-view composite graph convolutional network)	FC, high-order functional connectivity	fMRI	ABIDE	HC: 327 ASD: 286	Disease classification	ABIDE dataset: accuracy: 0.752	CMV-CGCN’s main architecture uses 2 parallel graph convolutional networks (GCNs) to process functional connectivity and high-order functional connectivity data.
17	Wang et al. [25]	CGL (contrastive functional connectivity graph learning)	FC	fMRI	ADHD-200	ADHD: 596	Disease classification	ADHD dataset: accuracy: 0.67	The main network architecture is a 2-layer GCN. It takes functional connectivity graphs as input and learns embeddings that are used in a contrastive learning framework for population-based fMRI classification.
18	Peng et al. [97]	GATE (graph CCA for temporal self-supervised learning)	FC	fMRI	ABIDE, FTD	HC: 544 ASD: 485 FTD: 95	Disease classification	ABIDE dataset: accuracy: 0.637 FTD dataset: accuracy: 0.724	GATE’s main architecture is a 2-layer GCN that is pretrained using a self-supervised learning approach based on temporal canonical correlation analysis.
19	Mahmood et al. [98]	MILIC (whole mutual information local to context)	Dynamic functional network features, FC	fMRI	HCP, FBIRN, ABIDE, OASIS	HC: 1,200 schizophrenia: 311 ASD: 569 AD: 372	Disease classification	For simulation data: AUC: 0.80–0.90 For real data (FBIRN dataset): AUC: around 0.87–0.90​	MILIC’s main architecture is a 3D CNN with 3 convolutional layers and 2 fully connected layers, used for learning from fMRI time series.
20	Jaiswal et al. [99]	CNN + LSTM (spatiotemporal encoder with contrastive learning)	BOLD	fMRI	BOLD5000, in-house dataset	HC: 24 TBI: 30	Disease classification	Accuracy: 0. 868	The main network architecture is a spatiotemporal encoder that combines 3D CNNs and LSTM networks. The 3D CNN part is used to extract spatial features from the 4D fMRI scans, and the LSTM part is used to model the temporal dynamics of these features.
21	Zhou et al. [100]	SGCN (sparse interpretable graph convolutional network)	Multi-modal node features, weighted brain connectivity graph	Multi-modal brain imaging (VBM-MRI, FDG-PET, AV45-PET)	ADNI	HC: 172 MCI: 471 AD: 96	Disease classification	ADNI dataset: accuracy: 0.826	The main network architecture is a 3-layer GCN designed for multi-modal brain network data. It incorporates a sparsity mechanism to learn interpretable regional and connective importance for Alzheimer’s disease diagnosis.

**Table 3.** Literatures of brain activity decoding using generative-contrastive learning in brain disorders

Index	References	SSL method	Data type	Data Modality	Dataset	Sample size	Downstream tasks	Performance metrics	Model architecture
22	Yang et al. [55]	BrainMass	BOLD	fMRI	ABIDE, HCP, UKB, ADHD-200, REST-MDD, etc.	HC: 17,130 ASD: 528 ADHD: 1,258 MCI: 1,151 AD: 149	Disease classification	ABIDE-I dataset: accuracy: 0.727 ADNI dataset: accuracy: 0.765 PPMI dataset: accuracy: 0.8 ABIDE-II dataset: accuracy: 0.728 SchizoConnect: accuracy: 0.765	BrainMass utilizes a GAT as the core encoder within its Mask-ROI Modeling framework. The framework also includes a feature alignment (FA) component, which uses a projector network (likely an MLP) to align the learned features. The encoder, pretrained in this way, is then used for downstream brain disorder diagnosis tasks.
23	Gazzar et al. [101]	Graph-S4 (graph state space model)	BOLD, FC	fMRI	Rest-Meta-MDD, SRPBS, ABIDE	HC: 1,450 MDD: 1,010 ASD: 649	Disease classification	MDD dataset: accuracy: 0.792 ABIDE dataset: accuracy: 0.721	The main network architecture is a single Graph-S4 layer followed by a linear classification layer. Graph-S4 is a graph state space model that extends the sequence-based S4 model to process graph data and learn from node features while implicitly considering the graph structure.
24	Zhang et al. [53]	α-GCNGAN (graph convolutional network + GAN)	FC	fMRI	ABIDE	ASD: 402 HC: 464	Disease classification	MDD dataset: F1 score: 0.828	Graph encoder: Uses GCN to encode brain network features. Generator/decoder: Converts latent space into generated graphs using feedforward neural networks. Discriminators: Ensure the generated graphs match real data through adversarial learning.
25	Oh et al. [50]	GC-GAN (graph convolutional networks + class-aware discriminator)	FC	fMRI	REST-meta-MDD	HC: 228 MDD: 249	Disease classification	MDD dataset: accuracy: 0.668	a-GCNGAN employs an architecture consisting of a GCN as the generator, another GCN as the discriminator, and a GCN-based autoencoder to generate synthetic brain network data.
26	Yang et al. [51]	GraphGAN++ (graph convolutional networks + GAN + multi-graph clustering)	FC	fMRI	ABIDE	HC: 468 ASD: 403	Disease classification	ABIDE dataset: accuracy: 0.705	A GraphGAN-based model generates realistic brain networks by preserving global distribution and local topology. It uses a WGAN to stabilize training, prevents mode collapse, and incorporates topology and latent space constraints

### Neurodegenerative disorders

As research into AD deepens, understanding how functional brain networks deteriorate with disease progression has become increasingly critical. Traditional static modeling [56,57] approaches often fall short in fully capturing these dynamic changes, as they provide only a snapshot of network states rather than their evolution over time. In contrast, advanced dynamic modeling techniques offer a novel perspective by tracking the temporal progression of network alterations, shedding light on the underlying mechanisms of neurodegeneration. Building on this, the dynamic modeling strategy of DynaMorph [45] effectively captures the progressive deterioration of functional brain networks in AD patients. Specifically, it identifies distinct temporal patterns of network disruption, including the sequential weakening of long-range connectivity and the emergence of localized network instability. These findings provide deeper insights into how the pathological features of AD evolve over time, potentially illuminating critical windows for therapeutic intervention. Similarly, DGCL [29] applies a graph-based self-supervised contrastive learning framework to identify 17 critical ROIs associated with AD, including the frontal lobe, precuneus, paracentral lobule, superior frontal gyrus, and caudate nucleus. This framework leverages node-level representations by maximizing agreement between augmented views of the same brain region while minimizing similarity across different regions. The reconstructed brain networks highlight pathological disruptions in AD, such as weakened long-range FC (e.g., between the frontal lobe and precuneus) and increased segregation within local subnetworks, reflecting impaired global integration and abnormal local clustering. The masking learning mechanism of ST-MAE [58] leverages a spatiotemporal masked autoencoding approach to dynamically reconstruct FC networks. In the context of Parkinson’s disease (PD), ST-MAE effectively identifies disrupted connections between the basal ganglia and cortex, as well as abnormalities in cortico-striatal and cortico-thalamic pathways, which facilitates the identification of specific biomarkers, such as weakened interregional connectivity strength and altered clustering coefficients, which are indicative of disease progression and severity. Single-photon emission computed tomography (SPECT) imaging parameters, including striatum asymmetry, putamen-to-caudate ratios, and regional uptake values, were combined with clinical variables such as age, biomarkers, and symptom profiles to construct multimodal graph representations. Through the application of co-attention mechanisms, alignment between imaging-derived and clinically derived feature spaces was achieved, enabling the extraction of shared embeddings. As a result, the multimodal contrastive cross-view graph learning framework [59] approach facilitated the delineation of distinct clusters specific to PD patients. Insights gained from these embeddings have revealed not only the heterogeneity in patient-specific pathological variations but also the dopaminergic dysfunction underlying disease progression, providing a foundation for tailored therapeutic strategies in the future.

### Mental disorders

The CMV-CGCN framework [27] has identified distinct and quantifiable patterns of abnormal FC in the brains of individuals with autism spectrum disorder (ASD), which includes disrupted correlations between specific brain regions and higher-order interactions that contribute to the unique FC profiles associated with ASD. Moreover, the synergistic integration of FC and HOFC highlights the hierarchical organization and intricate interactions within the brain networks of ASD patients. GraphGAN++ [51] effectively identifies biologically meaningful functional subnetworks and detects subtle abnormalities in the brain networks of ASD patients. Through multi-graph clustering based on resting-state brain FC, this model reveals ASD-specific network reorganization, including disrupted inter-regional connectivity and weakened local network integration. The identified subnetworks are consistent with previous neuroimaging studies [60–62], reinforcing the validity of ASD-specific biomarkers. Notably, some specific aberrant functional connections are directly associated with core ASD symptoms, including deficits in social interaction and repetitive behaviors. GCN_SSL [63] effectively monitors long-term dynamic shifts in brain functional networks during ASD symptom progression, pinpointing time-dependent connectivity changes in brain areas linked to social behavior, such as fluctuations in the FC of the amygdala, hippocampus, and fusiform gyrus. These changes are crucial for understanding social cognition and emotion regulation in the developmental trajectory of ASD, providing insights into the underlying neural mechanisms and potential biomarkers. To tackle the heterogeneity of attention-deficit/hyperactivity disorder (ADHD), BrainMass [55] has successfully pinpointed crucial biomarkers linked to temporal fluctuations in ADHD-related brain network connectivity. This research underscores the significance of subnetworks like the default mode and control networks, which exhibit altered interactions in individuals with ADHD. Besides, the DVAE [44] facilitates the creation of functional brain networks from resting-state fMRI data, enabling the identification of potential ADHD biomarkers. These biomarkers, manifested in unique FC patterns, serve as a foundation for differentiating individuals with ADHD from healthy controls and offer a deeper understanding of the disorder’s neurobiological underpinnings. In summary, SSL models encode complex neuroimaging data into a latent space, enabling the extraction and analysis of key neurofunctional features within a clinically relevant context. This approach has revealed distinctive biomarkers linked to attention deficits and impulsivity, which aids in the more accurate detection of ADHD subtypes and supports early diagnostic forecasting.

### Other neurological diseases

For the accurate identification of epileptic foci, MBrain [64] reveals unique spike-and-wave discharge signatures during seizures by explicitly capturing the intrinsic interplay between spatial and temporal aspects of brain activity​. Notably, this SSL model demonstrates robust cross-patient generalizability, as validated through extensive experiments on varied datasets encompassing both SEEG and EEG signals, highlighting its effectiveness in real-world applications. Furthermore, spatiotemporal encoder with contrastive learning (CNN + LSTM) enables the effective extraction of neural features from unlabeled data in traumatic brain injury (TBI) patients [65]. Moreover, this approach can identify multiple brain regions that contribute to cognitive fatigue (CF), potentially delineating a fatigue network. The brain regions tested using the Chaudhuri model of CF and other fatigue-related brain areas are caudate, anterior insula, medial prefrontal cortex, and middle frontal gyrus [66]. The activation patterns in these regions reveal the neurobiological basis of CF, and the model demonstrates superior accuracy in predicting self-reported CF scores compared to traditional methods, particularly beneficial in the rehabilitation of brain injury patients [67].

## Applications of SSL in Identifying Disease Biomarkers

In the realm of brain network analysis for brain disorders, SSL offers a powerful approach for identifying disease markers. Typically, SSL methods achieve this by leveraging learned representations from unlabeled data to rank features at the ROI level or connectivity level. This ranking is based on the features’ contributions to classification tasks, which are often quantified through ranking scores derived from feature importance measures in downstream models. For instance, graph-based SSL studies have identified critical ROIs and connectivity patterns associated with disorders like AD [68–73] and ASD [68,74–78], findings that align with prior neurobiological research. However, a notable limitation emerges from the reliance on public datasets, which are often cross-sectional in design and lack follow-up visits involving disease-targeted interventions. This limit hinders the validation of identified disease markers derived from SSL studies, as it remains unclear whether they accurately reflect disease progression or can effectively guide therapeutic outcomes. Despite this challenge, intriguing insights have emerged from multimodal SSL approaches. For example, previous studies [70,79,80] demonstrated that an SSL model trained on multimodal neuroimaging data can enhance the biological plausibility of the identified biomarkers, compared with single modality. This suggests that multimodal SSL may provide a more comprehensive view of brain alterations, potentially improving the robustness and clinical relevance of disease marker identification.

## Conclusion and Future Perspectives

Over the past few years, the rapid release of multisite and multimodal neuropsychiatric brain functional neuroimaging data has catalyzed remarkable advancements in SSL frameworks for the detection and prediction of brain disorders. This systematic review endeavors to offer a comprehensive overview of these methods across diverse medical scenarios. In the subsequent sections, we will provide a summary of the strengths and limitations of the current SSL models in the context of brain disorders, as well as discuss potential future opportunities.

### Advantages and challenges

First, SSL enables consistent multimodal alignment and fusion in neuroimaging data, as well as cross-modal generation. This is particularly crucial in studying brain disorders, which often manifest across multiple neuroimaging modalities. For example, SSL demonstrates effective integration of misaligned and unpaired multimodal neuroimaging data through the use of generative tasks, resulting in superior stability and improved performance in disease detection. In the context of AD, for instance, SSL can effectively fuse structural MRI, functional MRI, and EEG data to provide a more holistic view of the disease pathology, potentially leading to earlier and more accurate diagnosis. By generating high-quality synthetic data, SSL can also enhance neuroimage datasets and bolster disease discriminative model performance in scenarios of data scarcity [81] and missing modalities [82]. This is especially beneficial for rare brain disorders where patient data are inherently limited. Second, in clinical domains characterized by a scarcity of task-specific neuroimaging data, SSL offers considerable benefits. By leveraging self-supervised tasks like contrastive learning and masked modeling, SSL enables models to extract generalized features from unlabeled brain activity, showing promise for few-shot or even zero-shot learning scenarios. This is highly relevant to brain disorder research, where obtaining large, labeled datasets for specific patient populations can be challenging and ethically complex. These latent neural representations effectively captured by SSL models may exhibit strong transferability across diverse brain diseases. For example, with only 20% annotated samples, SSL model can outperform the supervised learning model in differentiating between ADHD and isolated rapid eye movement sleep behavior disorder (iRBD) [83], highlighting its great potential for clinical applications where annotated samples are limited. Extending this, SSL models trained on large, unlabeled datasets from healthy controls and individuals with various brain disorders could potentially be fine-tuned with limited labeled data to diagnose new patients or even predict disease progression in conditions like PD. However, the heuristic nature of pretraining task design poses a significant challenge to this transferability. For instance, masked modeling in SSL, such as reconstructing masked brain regions, may not fully align with downstream tasks like disease classification that require discriminative features rather than comprehensive reconstructions [9]. This misalignment can limit the model’s ability to prioritize disease-specific biomarkers, such as altered connectivity in ADHD or iRBD, over general brain activity patterns. Addressing this requires moving beyond manual task design toward automated strategies, like neural architecture search, to optimize pretraining objectives for specific clinical tasks, ensuring robust feature extraction tailored to brain disorder heterogeneity. In contrast, contrastive learning may offer a more suitable alternative for disease classification tasks due to its focus on discriminative feature extraction. Methods like SimCLR [17] and MoCo [84] optimize class invariance by distinguishing similar and dissimilar samples [9], making them adept at identifying subtle differences in brain activity patterns critical for disorders like ADHD or PD. While our generative and generative-contrastive approaches excel in data augmentation under scarcity, their reconstruction bias may dilute classification performance compared to contrastive methods, underscoring a trade-off between generative robustness and discriminative precision in clinical applications.

Third, the implementation of SSL process can provide novel biological insights for neuroimaging-based disease detection by adeptly capturing the internal spatiotemporal dependencies inherent in brain activities. This ability to uncover hidden patterns is particularly valuable for understanding the complex pathophysiology of brain disorders. Identification of disease-specific functional biomarkers can be facilitated by employing either contrastive multi-view learning, which ranks FC-wise contributions [85], or region-aware graph attention mechanisms [86], utilizing a masked auto-encoder module [42]. For example, using SSL, researchers might identify novel FC patterns that are specifically disrupted in schizophrenia, potentially leading to new therapeutic targets focused on restoring these dysfunctional networks.

It is crucial to underscore the current limitations of SSL when applied to brain network applications. First, SSL approaches often encounter difficulties in providing biologically meaningful interpretations for model decisions. This issue is particularly pertinent in the field of neuroimaging, where understanding the underlying biological mechanisms is crucial for translating SSL model outputs into actionable clinical insights. To address this, there is a growing need to integrate SSL with domain knowledge in neuroscience. For instance, incorporating known brain network architectures or established neurobiological pathways as constraints in SSL models could improve the interpretability of learned features in the context of depression or anxiety disorders. This could involve using prior biological insights to guide the learning process, ensuring that the model’s features align with known neurobiological processes. Second, there is ongoing debate about the extent to which synthetic datasets generated by GAN-based SSL models accurately mirror the characteristics of real-world brain networks. The inherent uncertainty in generative frameworks, particularly when dealing with unbalanced or sparse data distributions, raises concerns about the fidelity and reliability of these models. This is especially critical in brain disorder datasets, which can be highly heterogeneous and influenced by factors like medication, disease stage, and comorbidities. Generating realistic synthetic brain networks that capture this complexity, especially for conditions like ASD with its diverse clinical presentations, remains a significant hurdle. This limitation emphasizes the need for more robust evaluation metrics and validation processes. Specifically, evaluating synthetic data not only on statistical similarity but also on its ability to reproduce known disease-related patterns and biomarkers is crucial in brain disorder applications. Third, the multimodal integration encounters several limitations that impede the broader application of SSL in translational medical research. A key challenge is data heterogeneity, as different modalities often vary in spatial resolution, temporal dynamics, and noise characteristics, making it difficult to align and integrate these diverse data sources effectively. In the context of brain disorders, data heterogeneity can be further exacerbated by variations in acquisition protocols across different research sites or clinical settings, making it difficult to pool and analyze large-scale multimodal datasets for diseases like bipolar disorder or schizophrenia. For instance, the integration of EEG with fMRI data necessitates sophisticated algorithms to reconcile their intrinsic disparities. The computational complexity in this scenario poses a considerable challenge, especially for advanced models like hierarchical GCN and transformer-based architectures. These models require substantial computational resources, which can limit their scalability and real-time feasibility in clinical settings. Another critical concern is the missing modalities within multimodal SSL frameworks, where incomplete datasets are common in real-world scenarios. While reconstruction-based SSL models, exemplified by CD_SSL [87], endeavor to generate synthetic data to compensate for missing information, the fidelity and biological plausibility of these reconstructed features are frequently susceptible to noise and biases inherent to specific modalities. In brain disorder research, missing modalities can arise due to various reasons, including patient compliance issues or limitations of specific imaging techniques for certain populations (e.g., fMRI in patients with metallic implants). The reconstructed data for missing modalities, especially in sensitive contexts like pediatric brain disorders, need to be carefully validated for potential biases and artifacts. Overcoming these challenges necessitates the development of innovative approaches that adeptly reconcile computational efficiency, interpretability, and resilience to data scarcity or corruption, especially as multimodal integration techniques increasingly approach practical implementation within the realms of neuroscience and clinical practice.

### Future opportunities

In the pursuit of advancing SSL methodologies for brain disorders, it is imperative to encourage efforts aimed at addressing the transdiagnostic heterogeneity observed in psychological disorders, particularly those with overlapping symptoms. Advancements in SSL techniques, particularly those that capitalize on extensive unlabeled brain functional datasets, demonstrate great potential in discerning new subtypes of transdiagnostic psychosis. By capturing subtle reconfiguration of brain functional dynamics, these methods could pave the way for a deeper understanding of the pathophysiological mechanisms of psychosis, leading to more individualized treatment plans. Considering the critical role of data harmonization in addressing site-specific variances within multisite neuroimaging datasets, we advocate for the integration of this module into SSL frameworks by researchers. Furthermore, brain activity decoding by SSL models may present new opportunities to elucidate the functional brain mechanisms underlying cognitive states. Recent advancements in SSL have demonstrated remarkable effectiveness in reconstructing visual-semantic information from fMRI signals that are triggered by video stimuli [88,89]. Broadly speaking, we hypothesize that the integration of SSL-based models in decoding task-based brain functional recordings for neuropsychiatric patients can uncover novel insights underlying cognitive dysfunctions in brain disorders. Such models offer a promising pathway for bridging neuroscience and clinical practice, ultimately supporting the development of innovative therapeutic strategies. Another promising future direction lies in integrating nonimaging modalities into the SSL framework. For instance, the proposed video-audio-text transformer (VATT) model [90] demonstrates the ability of SSL to process raw video, audio, and text signals in parallel. It aligns these signals in hierarchical common spaces using noise contrastive estimation (NCE) and multiple instance learning NCE (MIL-NCE), achieving state-of-the-art performance. Similarly, in the medical context, clinical interview videos that capture patient visual and auditory cues (e.g., facial expressions and tone) could be tokenized and encoded alongside medical notes. This process could be potentially enhanced by domain-specific language models such as BioBERT [91] to improve medical textual understanding. The DropToken technique in VATT could also mitigate computational challenges associated with high-resolution video and lengthy notes. Future work could involve designing medical-specific pretraining tasks, such as aligning video segments of patient interviews with corresponding diagnostic descriptions in notes, and constructing a dataset of unlabeled medical multimodal data to validate this approach. This extension not only broadens the applicability of SSL in healthcare but also leverages the organic supervisory signals inherent in multimodal medical data, reducing reliance on costly annotations.
